# Effectiveness and safety of Janus Kinase inhibitors in patients with rheumatoid arthritis, results from a multicenter observational “real-life” GIRRCS (Gruppo Italiano di Ricerca in Reumatologia Clinica e Sperimentale) study

**DOI:** 10.1007/s10067-026-08373-8

**Published:** 2026-09-02

**Authors:** Daniela Iacono, Maura Raimondi, Valentina Marino, Flavia Riccio, Maria Laura Marchesano, Alessandra Milone, Addolorata Corrado, Cinzia Rotondo, Ilaria Firenze, Lidia La Barbera, Chiara Rizzo, Simona Montalbano, Luca Navarini, Alberto Lo Gullo, Marianna Oliva, Elvira Favoino, Ida Orlando, Francesco Paolo Cantatore, Paola Cipriani, Enrico Tirri, Roberto Giacomelli, Federico Perosa, Giuliana Guggino, Piero Ruscitti, Francesco Ciccia

**Affiliations:** 1 Department of Precision Medicine, Rheumatology Unit, University of Campania “Luigi Vanvitelli”, Naples, Italy; 2 Department of Medical and Surgical Sciences, Rheumatology Clinic, University of Foggia, Foggia, Italy; 3 Department of Health Promotion, Mother and Child Care, Internal Medicine and Medical Specialties, Rheumatology Section-″P. Giaccone″, University of Palermo, Palermo, Italy; 4 Department of Medicine and Post-Acute Care, Rheumatology Unit, “Ospedale del Mare” Hospital, Naples, Italy; 5 Fondazione Policlinico Universitario Campus Bio-Medico, Rome, Italy; 6 Research and Clinical Unit of Immunorheumatology, Department of Medicine and Surgery, Università Campus Bio-Medico Di Roma, Rome, Italy; 7 Rheumatology Unit, Papardo Hospital, Messina, Italy; 8 Rheumatic and Systemic Autoimmune Diseases Unit, Department of Interdisciplinary Medicine (DIM), University of Bari Medical School, Bari, Italy; 9 Frangipane Bellizzi” Hospital, Internal Medicine Unit, Ariano Irpino, Avellino, Italy; 10 Department of Biotechnological and Applied Clinical Sciences, University of L′Aquila, L′Aquila, Italy

**Keywords:** JAK inhibitors, Rheumatoid arthritis, Safety, Treatment

## Abstract

**Objectives:**

To evaluate the “real-life” effectiveness and safety of Janus kinase inhibitors (JAKis) in patients with rheumatoid arthritis (RA) and to analyze the impact of cardiometabolic comorbidities on these outcomes.

**Methods:**

Patients with RA treated with JAKis were evaluated for disease activity scores, patient-reported outcomes (PROs), and safety in a 12‑month multicenter observational study conducted within the GIRRCS (Gruppo Italiano di Ricerca in Reumatologia Clinica e Sperimentale). Patients were grouped and compared according to the presence of type 2 diabetes (T2D), cardiometabolic multimorbidity (defined as the simultaneous presence of two or more among high blood pressure, T2D, and/or dyslipidaemia), and age ≥ 65 years.

**Results:**

440 patients treated with JAKis were included (82.9% female; mean age 61.0 ± 11.0 years); 41.4% had high blood pressure, 39.0% obesity, 28.4% dyslipidaemia, 14.3% T2D, and 3.2% a history of cardiovascular events. A statistically significant improvement was observed in all disease activity measures and PROs over the follow-up period. These improvements seemed to be not influenced by the presence of T2D, cardiometabolic multimorbidity, or age ≥ 65 years. During follow-up, a cumulative adverse events incidence of 18.9% was reported; these were mostly mild and reversible, with no life-threatening events. Drug discontinuation due to adverse events occurred in 7.7% of assessed patients (34 discontinuations due to adverse events). Safety outcomes appeared to be not significantly affected by T2D, cardiometabolic multimorbidity, or age ≥ 65 years.

**Conclusions:**

In our “real-life” multicenter cohort in patients with RA, JAKis demonstrated effectiveness with an acceptable safety profile despite the relatively short follow-up and the lack of power to assess clinically relevant differences for rare events. The safety profile should therefore be interpreted with appropriate caution. The presence of T2D, cardiometabolic multimorbidity, and age ≥ 65 years did not appear to adversely influence treatment response or safety.

**Table:** 

**Key Points** • *TJAK inhibitors were effective in reducing disease activity and improving patient-reported outcomes in real-life patients with RA.* • *Type 2 diabetes, cardiometabolic multimorbidity, and older age did not appear to impair treatment effectiveness of JAK inhibitors in patients with RA.* • *Most adverse events of JAK inhibitors were mild, reversible, and infrequently led to treatment discontinuation.* • *These findings may support the use of JAK inhibitors in clinically complex real-life RA populations encountered in daily practice.*

## Introduction

In recent years, the therapeutic approach to rheumatoid arthritis (RA) has progressively improved allowing achievement and maintenance of remission or low disease activity within a treat‑to‑target therapeutic strategy, thus preventing joint damage and disability over time [1, 2]. Among the pathogenic mechanisms involved in RA, the Janus kinase (JAK)-signal transducer and activator of transcription (STAT) pathway may play a key pathogenic role [3, 4]. In fact, JAK‑STAT signaling is crucial for immune cell development, differentiation, and inflammatory responses, and its dysregulation contributes to the persistent inflammatory state observed in RA [3, 4]. On these bases, by inhibiting this pathway, JAK inhibitors (JAKis) are able to modulate the aberrant immune response underpinning the pathogenesis of RA [3–5]. Consequently, JAKis have emerged as targeted synthetic disease‑modifying antirheumatic drugs (tsDMARDs) and represent an effective therapeutic option for patients with RA [5]. However, despite their established efficacy in randomized clinical trials, many safety concerns have recently emerged [5, 6]. In particular, the increased risk of cardiovascular (CV) events and malignancies reported in the ORAL Surveillance trial [6] has raised questions regarding the administration of JAKis in clinical practice leading physicians to carefully assess CV risk factors, thromboembolic events, and malignancies when prescribing these drugs. Nevertheless, long‑term extension studies and registry data did not entirely confirm the findings of ORAL Surveillance, suggesting the need for further “real‑life” evidence to better clarify these safety issues [7, 8].

Considering the high burden of CV risk factors in RA, an increased susceptibility to both clinical and subclinical atherosclerosis has been described [9, 10]. This may result from the interaction between chronic systemic inflammation and traditional CV risk factors [9, 10]. In fact, coexisting CV risk factors may reflect a common pathogenic background or synergistically contribute to more severe clinical outcomes in RA [11, 12]. Moreover, an increasing body of evidence has increasingly pointed out a close relationship between RA, insulin resistance (IR), and type 2 diabetes (T2D) [13]. Pro‑inflammatory cytokines, which are overexpressed in RA, may promote pancreatic β‑cell apoptosis via mechanisms also comprising an aberrant JAK/STAT activation, thereby contributing to T2D development [14]. Interestingly, treatment with JAKis in RA patients with concomitant T2D has been associated with improvements in both IR and disease activity, suggesting a possible bidirectional benefit [15].

Based on this background, we conducted a multicenter “real‑life” study to evaluate the effectiveness and safety of JAKis in patients with RA. In addition, we explored potential differences in treatment response and safety according to the presence of T2D, cardiometabolic multimorbidity, and age ≥ 65 years.

## Methods

### Study design/settings/partecipants

In this multicenter, chart‑based observational cohort study, we evaluated patients with RA treated with JAKis, including tofacitinib (TOF), baricitinib (BAR), upadacitinib (UPA), and filgotinib (FIL). All patients included in the study had moderate-to-high disease activity at baseline according to routine clinical assessment and treatment eligibility criteria for initiation of JAKis. Patients were recruited from nine tertiary rheumatology centers involved in the GIRRCS (*Gruppo Italiano di Ricerca in Reumatologia Clinica e Sperimentale*) network in Italy. Clinical evaluations were performed at predefined time points: baseline (T0, treatment initiation) and at 3 (T3), 6 (T6), and 12 (T12) months of treatment, covering the time-period from 2020 to 2022. Assessed patients were adults (≥ 18 years) fulfilling the 2010 ACR/EULAR classification criteria for RA [16]. Regarding treatment allocation for JAKis, treatment decisions were made according to routine clinical practice and physician judgment at the participating centers. All patients at baseline were characterized by moderate-to-active disease and were eligible for treatment with JAKis.

The study was conducted in accordance with the ethical principles of the Declaration of Helsinki and the principles of Good Clinical Practice. Approval was obtained from the local Ethics Committee (*Comitato Etico Azienda Sanitaria Locale 1 Avezzano/Sulmona/L’Aquila*, L’Aquila, Italy; protocol numbers 000331/17 and 0218811/19).

### Variables collected

We collected demographic and clinical characteristics, including age, sex, smoking status, and comorbidities such as obesity, T2D, high blood pressure (HBP), dyslipidaemia, and major adverse CV events (MACE), defined as non‑fatal myocardial infarction, non‑fatal stroke, and CV death [17]. Additional variables included a history of thrombotic events, varicella zoster virus infection, and malignancies. Disease‑related characteristics were also recorded, including disease duration, autoantibody profile [rheumatoid factor (RF) and anti‑cyclic citrullinated peptide antibodies (ACPA)], and the presence of bone erosions. Information on previous treatment with biologic DMARDs (bDMARDs), concomitant therapy with conventional synthetic DMARDs (csDMARDs), and glucocorticoids (GCs) were collected. Disease activity was assessed at each visit by tender and swollen joint counts, disease Activity Score in 28 joints calculated with C‑reactive protein (DAS28‑CRP) and Simplified Disease Activity Index (SDAI) were calcutated accordingly. Patient‑reported outcomes (PROs) included patient global assessment of disease activity (PGA), physician global assessment (PhGA), and patient‑reported pain and fatigue, all measured using a 0–10 cm visual analogue scale. C-reactive protein and erythrocyte sedimentation rate values were not analyzed separately, as CRP was incorporated into the DAS28-CRP score and erythrocyte sedimentation rate was not systematically collected.

Adverse events (AEs) were systematically recorded at each follow-up visit and graded according to the Common Terminology Criteria for AEs (CTCAE) as mild-to-moderate, severe, or life-threatening. Serious AEs (SAEs) were defined according to standard pharmacovigilance criteria and included events resulting in death, life-threatening conditions, hospitalization or prolongation of hospitalization, persistent or significant disability, or other medically important conditions requiring urgent medical intervention. Based on previous evidence regarding the safety profile of JAKis [6], AEs of special interest included MACE, serious infections (defined as infections requiring hospitalization, intravenous antimicrobial therapy, or urgent medical intervention), deep venous thromboembolism, malignancies, herpes zoster, and death. Treatment discontinuations due to AEs were also recorded. For each adverse event, the type of JAKi, time-to-event, and clinical outcome were collected.

Furthermore, patients were stratified into subgroups according to: (i) presence or absence of T2D; (ii) presence or absence of cardiometabolic multimorbidity, defined as the concomitant presence of two or more among HBP, T2D, and dyslipidaemia; and (iii) age ≥ 65 years. The definition of cardiometabolic multimorbidity was adopted because this condition may represent a risk profile distinct from that of a single cardiometabolic comorbidity, as previously performed in both general and disease‑specific populations [18–20]. Obesity was not included because body mass index may inadequately reflect cardiometabolic risk in RA owing to disease-related alterations in body composition, including rheumatoid sarcopenia/cachexia. Previous CV disease was also excluded because of its low prevalence in the study population and because the adopted definition was intended to capture the burden of cardiometabolic risk factors rather than established CV outcomes. HBP was defined as persistently recorded systolic blood pressure > 130 mmHg and/or diastolic blood pressure > 80 mmHg, and/or the use of antihypertensive medication [21]. T2D was defined according to clinical chart data as fasting plasma glucose ≥ 126 mg/dL on two separate occasions, the presence of classic symptoms of hyperglycaemia with random plasma glucose ≥ 200 mg/dL (11.1 mmol/L), or the use of glucose‑lowering therapy [22]. Given the difficulties in lipid assessment in RA and the so‑called “lipid paradox,” dyslipidaemia was defined by the use of lipid‑lowering medications [23, 24].

### Statistical analysis

Descriptive statistics were initially used to summarize the collected data. Continuous variables were expressed as mean ± standard deviation while categorical variables were reported as percentages. The effectiveness of JAKis was primarily assessed by evaluating the overall 12‑month change in DAS28‑CRP, which was considered the main outcome measure. To account for the longitudinal structure of the data, an age‑ and sex‑adjusted linear mixed‑effects model was applied, including random intercepts and random slopes, and assuming an unstructured covariance matrix. This approach allowed assessment of within‑patient changes over time while accounting for inter‑individual variability. Covariate selection for the primary mixed-effects model was based on an a priori parsimonious approach. Age and sex were included as key baseline confounders, whereas additional clinical variables, including baseline disease activity, previous bDMARD exposure, concomitant therapies, and comorbidity burden, were not incorporated into the primary model because they may lie, at least in part, along the causal pathway between treatment selection and clinical outcomes. Adjustment for such variables could introduce overadjustment bias and potentially attenuate treatment-related associations. Thus, no additional variables were added in this model considering the “real-life” study design, the cohort sample size, and to avoid a possible overadjustment bias due to the lack of statistical power. Nevertheless, residual confounding due to unmeasured or non-adjusted factors cannot be completely excluded. Other clinimetric outcomes were analyzed by ANOVA test, accounting for the longitudinal study design, the continuous nature of the variables, and multiple comparisons. McNemar test has been employed to test changes in rate of concomitant GCs among the onset and different time-point of observation. In addition, tentative and exploratory t-tests were performed by comparing disease activity indices at each follow‑up time point with baseline values to evaluate changes over time. Thus, to account for multiplicity across repeated follow-up assessments, the analyses of secondary outcomes were conducted using a longitudinal modeling approach with adjustment for multiple comparisons. Subsequently, comparisons between individual follow-up visits and baseline were performed as exploratory analyses to facilitate clinical interpretation of temporal response patterns. These latter analyses were considered descriptive and hypothesis-generating, and therefore were not used as the basis for the primary study conclusions. Furthermore, with exploratory purposes, comparisons were performed between subgroups of patients with and without T2D, with and without cardiometabolic multimorbidity, and patients aged ≥ 65 years and others. Continuous variables were compared using t-tests whereas categorical variables were compared using the chi‑squared test. These assessments were performed to evaluate the potential differences across predefined groups. Given the exploratory nature of these analyses, direct analyses were conducted without accounting for time and repeating comparisons at individual time points. Furthemore, incident cases of AEs were reported as cumulative incidence proportion, counting all events (also possible multiple events within the same patient), and incidence rate per person-years at risk, censoring the follow‑up at treatment discontinuation. Patients characterised by significant missing data in main endpoints were excluded from the analysis. A two‑sided P value < 0.05 was considered statistically significant.

All statistical analyses were conducted using the Statistical Package for the Social Sciences (SPSS), version 17.0 (SPSS Inc., Chicago, IL, USA).

## Results

### Baseline descriptive features of the whole cohort of assessed patients with RA and treated by JAKis

**Fig. 1:**
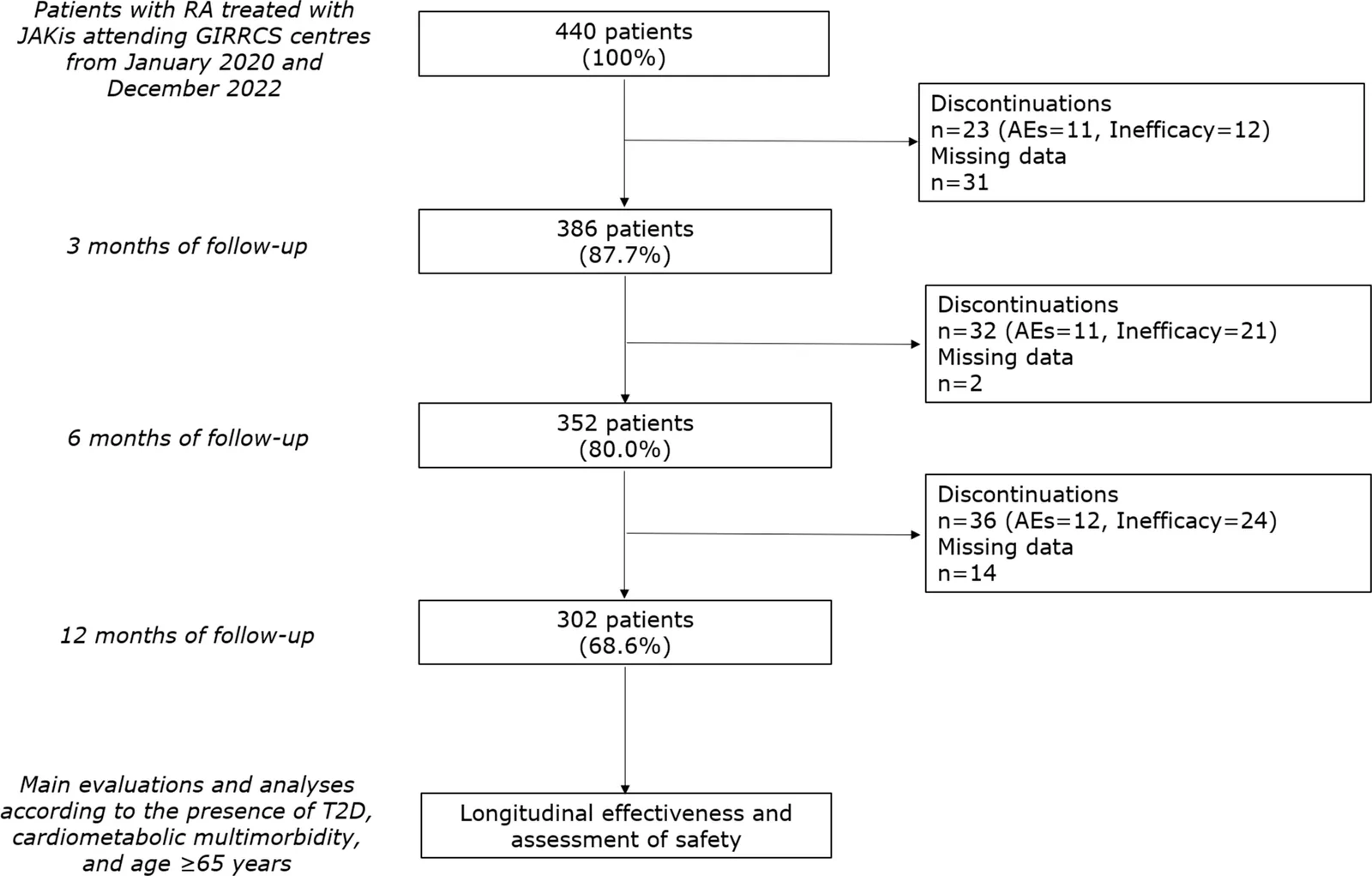
Study flow chart. In this figure, the flow chart of the study is reported. The number of patients contributing data at each time point is reported to facilitate interpretation of outcome analyses and to provide a transparent overview of patient retention throughout the study

Four hundred and forty patients were evaluated in a 12-month observational chart-based study and treated with JAKis, as reported in Fig. 1**.** All patients screened for eligibility were included in the study, and no patients were excluded because of missing data at baseline. Consequently, the number of screened patients corresponded to the number of assessed patients in the final study cohort. The majority of patients were females (82.9%, age 61 ± 11 years). Moreover, 144 patients (32.7%) were smokers. In our cohort, the rate of HBP was 41.4% (182 patients), obesity 39.0% (172 patients), dyslipidemia 28.4% (125 patients), T2D 14.3% (63 patients), history of MACE 3.2% (14 patients), respectively. Furthermore 10 patients had history of thrombotic events (2.3%), 11 patients had history of varicella zoster virus infection (2.5%), 16 patients had history of malignancies (3.6%), respectively. Moreover, the average duration of disease was 13.4 ± 9.9 years, RF was positive in 293 patients (66.6%), ACPA in 248 patients (56.4%). Based on distribution of disease activity at baseline, around one-third of patients were classified as having moderate disease activity (147/440, 33.4%), whereas two-thirds were classified as having high disease activity (293/440, 66.6%).

The rate of bone damage was observed in 56.8% (250 patients); 191 patients were bDMARD naive (43.4%), while 212 patients (48.2%) failed at least 2 bDMARDs. Finally, at baseline, 201 patients (45.6%) were treated with MTX (mean dosage 15 mg weekly), while 266 patients (60.4%) were concomitantly treated with GC therapy (mean dosage equivalent to 5 mg prednisone daily). These data are reported in Table 1**.** Furthermore, at baseline, the distribution of JAKis was as follows: 94 patients treated with TOF 10 mg daily (53 bDMARD-naïve); 188 patients with BAR 4 mg daily (86 bDMARD-naïve), 91 patients with UPA 15 mg daily (22 bDMARD-naïve), 67 patients with FIL 200 mg daily (27 bDMARD-naïve).

**Table 1:** Demographic and clinical features of the evaluated participants with rheumatoid arthritis, whole cohort

Patient number	440
Mean age, mean ± SD years	61.0 ± 11.0
Gender (female/male), n	365 F/75 M
Smoking habit, %	144/440 (32.7%)
High blood pressure, %	182/440 (41.4%)
Obesity, %	172/440 (39.0%)
Dyslipidemia, %	125/440 (28.4%)
T2D, %	63/440 (14.3%)
MACE, %	14/440 (3.2%)
Thrombosis	10/440 (2.3%)
History of herpes zoster	11/440 (2.5%)
History of malignancy	16/440 (3.6%)
Disease duration, mean ± SD, years	13.4 ± 9.9
Rheumatoid factor, %	293/440 (66.6%)
ACPA, %	248/440 (56.4%)
Bone damage, %	250/440 (56.8%)
bDMARD Naive, %	191/440 (43.4%)
≥ 2 previous bDMARDs, %	212/440 (48.2%)
JAKi Monotherapy, %	239/440 (54.3%)
Concomitant MTX%	201/440 (45.6%)
Concomitant GCs, %	266/440 (60.4%)
Mean SDAI baseline, mean ± SD	19.8 ± 4.38
Mean DAS28-CRP baseline, mean ± SD	3.9 ± 1.4

*SD* standard deviation, *T2D* type 2 diabetes, *MACE* major adverse cardiovascular events, *ACPA* Anti-Citrullinated Protein Antibodies, *bDMARD* biologic disease‑modifying antirheumatic drugs, *JAKi* janus kinase inhibitor, *MTX* methotrexate, *GCs* glucoicorticoids, *SDAI* Simplified Disease Activity Index, *DAS28-CRP* Disease Activity Score 28 joint count with C-Reactive Protein

### Effectiveness

**Fig. 2:**
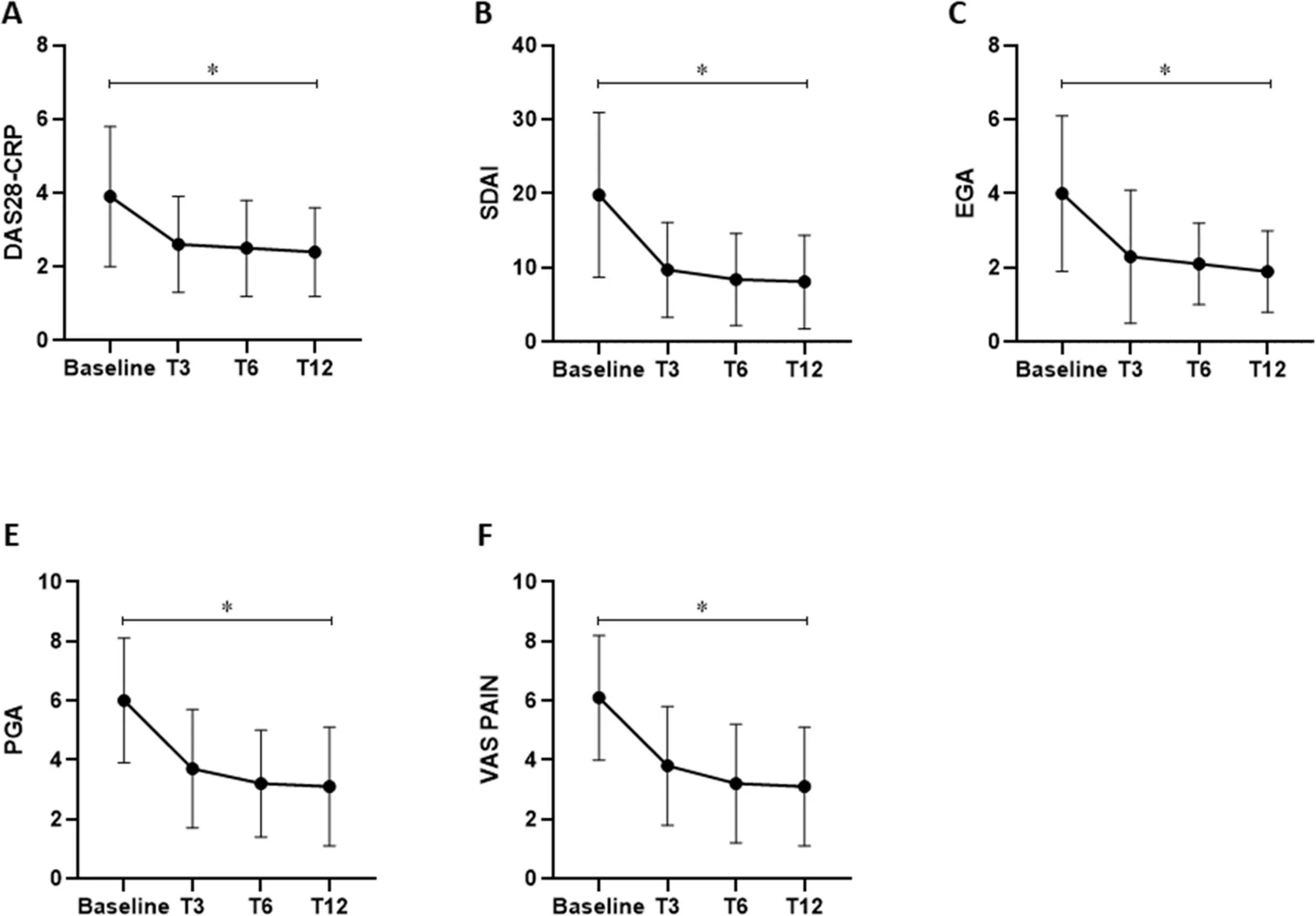
Main assessed clinimetric scores in patients treated with JAKis. Longitudinal changes in clinimetric outcomes during follow-up. Analyses were performed using repeated-measures ANOVA, accounting for the longitudinal study design, the continuous nature of the variables, and multiple comparisons. Results are presented as mean ± standard deviation (SD), and error bars indicate SD. (**A**) Mean DAS28-CRP during follow-up; (**B**) mean SDAI during follow-up; (**C**) mean Physician Global Assessment (PhGA) during follow-up; (**D**) mean Patient Global Assessment (PGA) during follow-up; (**E**) mean pain Visual Analogue Scale (VAS) during follow-up

A significant effectiveness of JAKis was observed in our cohort on the overall 12-month reduction of DAS28-CRP after adjusting the linear mixed model for age and male sex (β − 1.48, p = 0.011, 95% CI − 3.12 to − 0.89). In addition, the other clinimetric scores progressively and significantly reduced during the follow-up (*p* < 0.0001 for all scores) using a modelling approach that accounted for multiple comparisons across follow-up assessments. Visit-specific comparisons versus baseline were performed as exploratory analyses to provide a more granular characterization of temporal response patterns and should be interpreted as supportive rather than confirmatory findings. Thus, assessing individually each timepoint, three months after beginning treatment with JAKi, a statistically significant improvement was observed in all evaluated clinimetric scores and PROs and was confirmed during the subsequent evaluations. In particular, singularly comparing each time-point with baseline, we observed a statistically significant difference in mean DAS28-CRP at T3 (2.6 ± 1.3), T6 (2.5 ± 1.3), T12 (2.4 ± 1.2), when compared with baseline (baseline mean DAS28-CRP (3.9 ± 1.4) (*p* < 0.0001) Fig. 2A; a statistically significant difference was also observed for SDAI score at 3 months (T3) (9.7 ± 8.4), 6 months (T6) (8.4 ± 8.2), 12 months (T12) (8.1 ± 8.3), when compared with baseline (baseline mean SDAI 19.8 ± 11.1) (*p* < 0.0001). Figure 2B

Moreover, a statistically significant difference was observed in mean PhGA at T3 (2.3 ± 2.08), T6 (2.1 ± 2.2), T12 (1.9 ± 2.1), when compared with baseline (baseline PhGA 4.0 ± 3.1 (p < 0.0001) Fig. 2C**;** and mean PGA at T3 (3.7 ± 2.2), T6 (3.2 ± 2.3), T12 (3.1 ± 2.5), when compared with baseline (baseline mean PGA 6.0 ± 2.1 (*p* < 0.0001). Figure 2D

A statistically significant difference was also observed for VAS PAIN at T3 (3.8 ± 2.4), T6 (3.2 ± 2.4), T12 (3.07 ± 2.5), when compared with baseline (baseline mean PAIN VAS 6.1 ± 2.1 (*p* < 0.0001) Fig. 2E, and mean VAS FATIGUE at T3 (4.5 ± 2.4), T6 (4.0 ± 2.6), T12 (3.6 ± 2.7), when compared with baseline (baseline mean VAS FATIGUE 6.2 ± 2.3 (*p* < 0.0001).

Moreover, a statistically significant difference was observed in mean tender joint count at T3 (3.0 ± 3.8), T6 (3.2 ± 4.4), T12 (3.0 ± 4.6), compared to baseline (baseline mean 8.0 ± 5.9 (*p* < 0.001) and mean swollen joint count at T3 (1.0 ± 1.7), T6 (1.0 ± 1.5), T12 (0 ± 1.3), when compared with (baseline mean 3.0 ± 3.4 (*p* < 0.0001).

Finally, after 3, 6, and 12 months, the percentage of patients on GC treatment was 47.3%, 39.7%, and 36.4%, respectively, significantly lower when compared with the percentage at baseline of 60.4% (p < 0.0001 for all comparisons between assessed time-points and baseline).

### Clinical evaluation of patients with RA and treated by JAKis according to the presence of T2D, cardiometabolic multimorbidity, and aged ≥ 65 years

Furthermore, exploratory analyses were conducted to assess potential differences between predefined patient subgroups, specifically patients with and without T2D, patients with and without cardiometabolic multimorbidity, and patients aged ≥ 65 years compared with those younger than 65 years. These subgroup analyses were undertaken to explore the consistency of treatment responses across clinically relevant patient populations and to generate hypotheses for future studies. Given their exploratory nature, no adjustment for multiple comparisons was applied; therefore, the results should be interpreted with caution and regarded as supportive rather than confirmatory evidence.

Comparing patients with (*n* = 63) and without T2D (*n* = 377), statistically significant differences were found for age (mean age ± SD: 65.0 ± 5.0 years vs 60.0 ± 6.0 years, *p* < 0.0001), HBP (45/63 (71.4%) vs 137/377 (36.3%), *p* < 0.0001) and percentage of patients naïve to treatment with bDMARDs (36/63 (57.1%) vs 155/377 (41.1%), *p* = 0.017).

No statistically significant differences were found for disease activity during follow-up**,** as reported in Table 2**.** Comparing patients with (*n* = 100) and without (*n* = 340) cardiometabolic multimorbidity, statistically significant differences were found for age (mean age ± SD: 66.0 ± 11.0 years vs 60.0 ± 11.0 years, *p* < 0.0001) and percentage of patients naïve to treatment with bDMARDs (55/100 (55%) vs 136/340 (40%), *p* = 0.008). No statistically significant differences were found for disease activity during follow-up, as summarized in Table 3**.**

**Table 2:** Demographic and clinical features of the evaluated patients with RA grouped and compared according to the presence of T2D

	63 Patients with T2D	377 patients without T2D	*p*
Mean age, years ± SD	65.0 ± 5.0	60.0 ± 6.0	< 0.0001*
Gender (female/male), %	48/15 (76.2%)	317/60 (84.1)	0.12
Smoking habit, %	27/63 (42.8%)	117/377(31.0%)	0.06
Obesity, %	30/63 (47.6%)	142/377 (37.6%)	0.13
High blood pressure, %	45/63 (71.4%)	137/377 (36.3%)	< 0.0001*
Dyslipidemia, %	19/63 (30.1%)	106/377 (28.2%)	0.76
MACE, %	4/63 (6.3%)	10/377 (2.6%)	0.12
Thrombosis	2/63 (3.17%)	8/377(2.1%)	0,59
Disease duration mean, years ± SD	14.2 ± 4.2	13.3 ± 19.7	0.78
Rheumatoid factor,%	40/63 (63.5%)	253/377(67.1%)	0.57
ACPA, %	39/63 (61.9%)	209/377 (55.4%)	0.34
Bone damage, %	34/63 (53.9%)	216/377 (57.3%)	0.61
bDMARD Naive, %	36/63 (57.1%)	155/377 (41.1%)	0.02*
JAKi Monotherapy, %	38/63 (60.3%)	201/377 (53.3%)	0.30
Concomitant MTX %	25/63 (39.7%)	176/377 (46.6%)	0.31
Concomitant GCs, %	33/63 (52.4%)	233/377 (61.8%)	0.16
Mean DAS28-CRP T0 (± SD)	4.1 ± 1.6	3.7 ± 1.3	0.32
Mean DAS28-CRP T6 (± SD)	2.7 ± 1.5	2.5 ± 1.2	0.75
Mean DAS28-CRP T12 (± SD)	2.5 ± 1.3	2.4 ± 1.5	0.85
Mean SDAI T0 (± SD)	21.5 ± 11.2	19.6 ± 11.3	0.22
Mean SDAI T6 (± SD)	8.4 ± 8.6	8.4 ± 8.2	0.99
Mean SDAI T12 (± SD)	8.4 (7.8)	8.1 ± 8.2	0.82
Adverse events%	14/63 (22.2%)	69/377 (18.3%)	0.18

*SD* standard deviation, *T2D* type 2 diabetes, *MACE* major adverse cardiovascular events, *ACPA* Anti-Citrullinated Protein Antibodies, *bDMARD* biologic disease‑modifying antirheumatic drugs, *JAKi* janus kinase inhibitor, *MTX* methotrexate, *GCs* glucoicorticoids, *SDAI* Simplified Disease Activity Index, *DAS28-CRP* Disease Activity Score 28 joint count with C-Reactive Protein

**Table 3:** Demographic and clinical features of the evaluated patients with RA grouped and compared according to the presence of cardiometabolic multimorbidity

	100 Patients with Cardiometabolic multimorbidity	340 Patients without Cardiometabolic multimorbidity	*p*
Mean age, years ± SD	66.0 ± 1.0	60.0 ± 11.0	< 0.0001*
Gender (female/male), %	81/19 (81.0%)	285/55 (83.8%)	0.61
Smoking habit, %	40/100 (40.0%)	104/340 (30.6%)	0.08
Obesity, %	42/100 (42.0%)	130/340 (38.2)	0.51
MACE, %	8/100 (8.0%)	6/340 (1.8%)	0.002
Thrombosis, %	2/100 (2.0%)	8/340 (2.35%)	0.84
Disease duration, mean, years ± SD	14.1 ± 10.0	13.1 ± 10.0	0.35
Rheumatoid factor,%	60/100 (60.0%)	233/340 (68.5%)	0.12
ACPA, %	55/100 (55.0%)	193/340 (56.8%)	0.75
Bone damage, %	50/100 (50.0%)	200/340 (58.8%)	0.12
bDMARD Naive, %	55/100 (55.0%)	136/340 (40.0%)	0.008*
JAKi Monotherapy, %	58/100 (58.0%)	181/340 (53.2%)	0.4
Concomitant MTX, %	42/100 (42.0%)	159/340 (46.8%)	0.4
Concomitant GCs, %	59/100 (59.0%)	207/340 (60.8%)	0.75
Mean DAS28-CRP T0 (± SD)	3.9 ± 1.8	3.8 ± 1.5	0.54
Mean DAS28-CRP T6 (± SD)	2.7 ± 1.8	2.5 ± 1.2	0.69
Mean DAS28-CRP T12 (± SD)	2.5 ± 1.5	2.5 ± 1.7	0.93
Mean SDAI T0 (± SD)	21.5 ± 11.2	19.6 ± 11.3	0.22
Mean SDAI baseline (± SD)	21.1 ± 11.4	19.5 ± 11.3	0.22
Mean SDAI T6 (± SD)	8.2 ± 8.1	8.4 ± 8.2	0.85
Mean SDAI T12 (± SD)	8.0 ± 8.1	8.2 ± 8.3	0.86
Adverse events, %	21/100 (21.0%)	62/340 (18.2%)	0.23

*SD* standard deviation, *MACE* major adverse cardiovascular events, *ACPA* Anti-Citrullinated Protein Antibodies, *bDMARD* biologic disease‑modifying antirheumatic drugs, *JAKi* janus kinase inhibitor, *MTX* methotrexate, *GCs* glucoicorticoids, *SDAI* Simplified Disease Activity Index, *DAS28-CRP* Disease Activity Score 28 joint count with C-Reactive Protein

Finally, we divided our cohort into 2 further groups based on age at baseline, patients with age ≥ 65 years (mean age 72.0 years ± 11.0) (*n* = 168) and patients under 65 years (mean age 55.0 ± 7.0) (*n* = 272). This is reported in Table 4**.** We observed that patients under 65 years were more female (86.2% vs 77.9%, *p* 0.028), with a shorter disease duration (mean disease duration 11.7 ± 8.9 SD vs 16.2 ± 9.9 SD, *p* < 0.0001) and with lower rate of bone damage (51.5% vs 65.5%, *p* = 0.004) compared with older age group. As expected, we also observed a higher prevalence of HBP (60.1% vs 29.7%, *p* < 0.0001) and higher prevalence of MACE (7.1% vs 0.73%, *p* = 0.0002) in patients over 65 years with statistically significant differences compared to patients under 65 years. Finally, no statistically significant differences were found for disease activity at baseline and during follow-up.

**Table 4:** Demographic and clinical features of the evaluated patients with RA grouped and compared according to the presence of age ≥ 65 years

	168 Patients aged ≥ 65 years	272 Patients aged ≤ 65 years	*p*
Mean age, years ± SD	72.0 ± 11.0	55.0 ± 7.0	< 0.0001*
Gender (female/male), %	131/37 (77.9%)	234/38 (86.0%)	**0.028**
Smoking habit, %	46/168 (27.4%)	98/272 (36.0%)	0.06
Obesity, %	71/168 (42.3%)	101/272 (37.1%)	0.28
High blood pressure, %	101/168 (60.1%)	81/272 (29.7%)	< 0.0001*
Dyslipidemia, %	53/168 (31.5%)	72/272 (26.5%)	0.26
MACE, %	12/168 (7.1%)	2/272 (0.7%)	0.0002*
Thrombosis	5/168 (3.0%)	5/272 (1.1%)	0.15
Disease duration mean, years ± SD	16.2 ± 9.9 yrs	11.7 ± 8.9 yrs	< 0.0001*
Rheumatoid factor,%	111/168 (66.1%)	182/272 (66.9%)	0.86
ACPA, %	92/168 (54.8%)	156/272 (57.3%)	0.61
Bone damage, %	110/168 (65.5%)	140/272 (51.5%)	0.004*
bDMARD Naive, %	79/168 (47.0%)	112/272 (41.2%)	0.23
Monotherapy, %	93/168 (55.3%)	141/272 (51.8%)	0.47
Concomitant MTX, %	75/168 (44.6%)	131/272 (48.2%)	0.46
Concomitant GCs, %	101/168 (60.1%)	165/272 (60.6%)	0.92
Mean DAS28-CRP T0 (± SD)	3.8 ± 1.5	3.9 ± 1.7	0.63
Mean DAS28-CRP T6 (± SD)	2.6 ± 1.7	2.5 ± 1.5	0.68
Mean DAS28-CRP T12 (± SD)	2.4 ± 1.6	2.4 ± 1.8	0.91
Mean SDAI baseline (± SD)	18.8 ± 11.0	20.4 ± 11.4	0.14
Mean SDAI T6 (± SD)	8.2 ± 8.2	8.4 ± 8.5	0.78
Mean SDAI T12 (± SD)	8.4 ± 7.8	8.1 ± 8.2	0.82
Adverse events %	39/168 (23.2%)	44/272 (16.2%)	0.06

*SD* standard deviation, *MACE* major adverse cardiovascular events, *ACPA* Anti-Citrullinated Protein Antibodies, *bDMARD* biologic disease‑modifying antirheumatic drugs, *JAKi* janus kinase inhibitor, *MTX* methotrexate, *GCs* glucoicorticoids, *SDAI* Simplified Disease Activity Index, *DAS28-CRP* Disease Activity Score 28 joint count with C-Reactive Protein

### Safety

For each adverse event, the type of JAKis, time-to-event, and clinical outcome were collected and reported in Table 5. During the 12‑month follow‑up period, a total of 83 AEs were recorded among the 440 patients included, corresponding to a cumulative incidence of 18.9%. Overall, 7.7% of patients discontinued the therapy of JAKis due to AEs. Drop‑out rates due to AEs were evaluated at each time point. At month 3 (T3), 386 patients were actively followed, while 23 discontinued the JAKis, 11 of whom were due to AEs. At month 6 (T6), 352 patients remained under follow‑up and 32 discontinued the JAKis, including 11 due to AEs. On month 12 (T12), 302 patients were actively followed and 36 discontinued the JAKis, 12 because of AEs, as reported in Table 5. The overall incidence rate of AEs was 17.6% (95% CI 17.2–18.0) per 100 person‑years, based on a total of 363 person‑years. The incidence rate of AEs leading to treatment discontinuation was 7.2% (95% CI 3.5–9.8) per 100 person‑years based on a total of 363 person‑years.

**Table 5:** Analytical description of side effects in assessed cohort of patients

	Special interest AEs			Mild-moderate AEs		
	*3 months*	*6 months*	*12 months*	*3 months*	*6 months*	*12 months*
*Tofacitinib*	-	-	-	2 infections (1 discontinuation); 1 herpes zoster; 1 laboratory abnormalities; 1 skin reaction (1 discontinuation); 1 gastrointestinal disorder; 1 patient intolerance (1 discontinuation)	1 infection (1 discontinuation); 1 herpes zoster	2 patient intolerances; 1 infection; 2 laboratory abnormalities (2 discontinuations)
*Baricitinib*	-	1 acute myocardial infarction (1 discontinuation)	1 leiomyosarcoma (1 discontinuation)	11 laboratory abnormalities (1 discontinuation); 2 skin reaction, 1 patient intolerance (1 discontinuation); 2 infections (1 discontinuation); 2 herpes zosters; 1 gastrointestinal disorder (1 discontinuation); 1 atrial fibrillation (1 discontinuation)	8 laboratory abnormalities (2 discontinuations); 1 skin reaction (1 discontinuation); 1 infection; 3 herpes zosters (2 discontinuations)	8 laboratory abnormalities (3 discontinuations); 1 infection; 2 herpes zosters (1 discontinuation); 1 superficial vein thrombosis; 1 gastrointestinal disorder
*Upadacitinib*	-	-	-	3 infections (1 discontinuation); 1 laboratory abnormalities (1 discontinuation); 1 herpes zoster (1 discontinuation)	3 laboratory abnormalities (1 discontinuation); 2 infections (2 discontinuations)	2 infections; 1 herpes zoster (1 discontinuation); 1 laboratory abnormalities (1 discontinuation)
*Filgotinib*	-	-	-	1 skin reaction	1 patient intolerance (1 discontinuation); 1 infection; 1 laboratory abnormalities	2 laboratory abnormalities (2 discontinuations); 1 patient intolerance (1 discontinuation)

We did not observe life-threatening AEs, cases of deep vein thrombosis, serious infections, or deaths. Two AEs of special interest were reported during the follow-up. Specifically, 1 MACE (myocardial infarction) and 1 case of leiomyosarcoma were reported. The remaining AEs, which were recorded during the study, were codified as mild to moderate. One patient developed atrial fibrillation and one superficial vein thrombosis. Laboratory abnormalities represented the most frequent AEs, occurring in 38 patients, mainly anemia and hypertransaminasemia; these were mild and reversible after drug discontinuation. Non‑serious infections were reported in 27 patients, including herpes zoster reactivation in 11 unvaccinated patients. Patients with herpes zoster were predominantly female, had a mean age of 61 years, and frequently presented cardiometabolic risk factors, including obesity (n = 3), HBP (*n* = 3), and dyslipidaemia (*n* = 3); only one patient was a smoker. Less frequent AEs included skin reactions (*n* = 5) and gastrointestinal disorders (*n* = 3), mainly occurring within the first three months of treatment. Six patients discontinued treatment because of poorly defined intolerance.

No significant differences in rates of AEs were found between patients with and without T2D or between patients with and without cardiometabolic multimorbidity (Tables 2 and 3). Although not significant, a higher proportion of AEs was observed in patients aged ≥ 65 years compared with others (23.2% vs 16.6%, *p* = 0.06); however, a MACE occurred in this subgroup (Table 4).

In Table 6, the reasons for treatment discontinuation according to individual JAKis and follow-up duration are reported.

**Table 6:** Reasons for treatment discontinuation according to JAK inhibitor and follow-up duration

Individual JAK inhibitor	Baseline, n	3 months, discontinuations *n* (%)	Due to inefficacy	Due to AEs	6 months, discontinuations *n* (%)	Due to inefficacy	Due to AEs	12 months, discontinuations *n* (%)	Due to inefficacy	Due to AEs
Tofacitinib	94	6 (6.4)	3	3	10 (10.6)	9	1	7 (7.4)	5	2
Baricitinib	188	10 (5.3)	5	5	11 (5.9)	5	6	18 (9.6)	13	5
Upadacitinib	91	3 (3.3)	0	3	6 (6.6)	3	3	4 (4.4)	2	2
Filgotinib	67	4 (6.0)	4	0	5 (7.5)	4	1	7 (10.4)	4	3

The low number of safety events observed during follow-up substantially limits the statistical power to detect differences across clinically relevant subgroups. Therefore, the absence of observed differences according to T2D status, cardiometabolic multimorbidity, or age should not be interpreted as evidence of comparable risk profiles. Rather, these findings should be considered descriptive and interpreted with caution, as the possibility of type II error cannot be excluded.

## Discussion

In this multicenter observational cohort study evaluating patients with RA treated with JAKis over a 12‑month period, we observed a rapid reduction in disease activity during follow‑up. The marked improvement in disease activity already evident after three months of therapy is consistent with the rapid mechanism of action of JAKis, which directly interfere with key pathogenetic pathways involved in RA [25, 26]. Overall, our findings may parallel previous studies confirming the effectiveness JAKis in RA [27, 28]. In addition to improvements in disease activity, we also observed a significant amelioration of PROs, including PGA, pain, and fatigue. These results may reflect the dual mechanism of action of JAKis, which modulates both inflammatory and nociceptive pathways, potentially leading to pain reduction beyond the control of inflammation alone [29–32].

The present findings are broadly consistent with evidence from European registries and real-world comparisons between JAKis and bDMARDs, which have generally confirmed the effectiveness of JAKis in routine clinical practice across diverse patient populations [33–37].

Data from large collaborative registries have shown that JAKis may provide clinical responses comparable to those observed with bDMARDs, with similar treatment retention and effectiveness outcomes after adjustment for baseline characteristics [33–37]. Nevertheless, direct comparisons across studies should be interpreted cautiously owing to differences in study design, patient characteristics, baseline risk profiles, and outcome definitions [33–37]. Notably, the effectiveness observed in older patients in our cohort aligns with previous reports evaluating both JAKis and bDMARDs, suggesting that clinically meaningful improvements in disease activity can be achieved even in elderly individuals [38–40]. While advancing age is frequently associated with a greater burden of comorbidities and a potentially increased risk of adverse outcomes, current real-world evidence indicates that age alone should not necessarily preclude the use of effective targeted therapies [38–40].

In our cohort, during follow-up, a cumulative incidence of AE in 18.9% of patients was reported due to JAKis; they were mostly mild and reversible, with no life-threatening events. Drug discontinuation due to AEs occurred in 7.7% of patients. In this context, comparative observational studies have not consistently identified a significant excess risk of MACEs, malignancies, or venous thromboembolism among appropriately selected patients treated with JAKis compared with bDMARDs [33–40]. However, differences in patient characteristics, treatment indications, and CV risk profiles across real-world cohorts should be carefully considered when interpreting these findings [33–40]. Our results should be cautiously contexualized against the safety outcomes highlighted in the ORAL Surveillance trial, which raised concerns when prescribing JAKis in daily practice. Although the mean age of our cohort was below the 65‑year threshold considered a risk factor for AEs (61.0 ± 11.0 years), only one MACE was observed during follow‑up. Taking into relevant account the short duration of follow‑up and with appropriate caution, the incidence of MACEs in our cohort appeared cautiously lower than what reported in ORAL Surveillance [6]. Furthermore, this finding may appear in line with what expected in “real‑life” settings and in the general population according to European Society of Cardiology data [41–43]. One malignancy (leiomyosarcoma) was also observed. Overall, these findings are consistent with long‑term extension studies and registry data which did not fully confirm the ORAL Surveillance safety signals [7, 8]. No cases of deep venous thromboembolism or serious infections were observed in our cohort. A low percentage of non‑serious infections was reported in our study, including herpes zoster reactivation. These were observed in unvaccinated patients, further supporting the current recommendations for vaccination against herpes zoster in patients treated with JAKis. Patients aged ≥ 65 years showed a higher rate of comorbidities and a higher rate of AEs; however, only one MACE occurred in this subgroup, in agreement with previously reported risk modifiers identified in post‑hoc ORAL Surveillance analyses [8, 42]. Cumulatively, less than 10% of patients discontinued JAKis due to AEs, with laboratory abnormalities and non‑serious infections being the most frequent, findings consistent with other real‑world studies [8, 42, 43]. However, the comparability between populations of our cohort and ORAL Surveillance trial is clearly limited in terms of risk enrichment, duration of follow‑up, event adjudication and statistical power. Our study was designed as a short‑term observational chart-based cohort which reported few “hard” events. Thus, the absence of an evident signal in our cohort study do not allow any refutation of ORAL Surveillance data, but rather provides complementary and only partially reassuring findings suggesting the need of additional specific designed and adequately powered studies to further reinforce our findings.

Recent “real‑life” evidence has suggested that cardiometabolic multimorbidity identifies a more severe clinical phenotype of RA [44, 45]. In our study, however, no significant differences were found in treatment effectiveness or safety among patients with T2D or cardiometabolic multimorbidity, despite these subgroups being older and presenting some clinical differences. Taking into account the very low incidence of major safety events, the study was not designed nor sufficiently powered to reliably evaluate subgroup-specific differences in rare adverse outcomes. Consequently, the lack of observed safety signals in patients with T2D, cardiometabolic multimorbidity, or older age should be interpreted cautiously, and the possibility of type II error should be acknowledged. Given the exploratory nature of these analyses, no formal longitudinal or time-dependent modelling was applied. Comparisons were therefore performed cross-sectionally, without accounting for time as a variable and repeating analyses at individual follow-up time points. Consequently, the results should be interpreted as hypothesis-generating rather than confirmatory. In addition, since specific correlation analyses between cardiometabolic risk factors and treatment response were outside the primary scope of this study, further specifically designed investigations are warranted to better elucidate these relationships.

This study has some limitations, including the observational design, the absence of a control group, and the relatively short follow‑up duration of 12 months, which may limit the generalizability of the results. Nonetheless, the multicenter “real‑life” design allowed the evaluation of a large and clinically heterogeneous patient population, potentially better reflecting daily clinical practice compared with randomized controlled trials. Patients were recruited exclusively from tertiary referral rheumatology centers, which may have introduced referral bias by enriching the cohort with individuals presenting more severe or treatment-refractory disease. Therefore, the generalizability of our findings to community-based RA populations may be limited. In addition, treatment allocation of JAKis was based on routine clinical practice and physician judgment, and potential variability in prescribing patterns across participating centers cannot be completely excluded. Another important interpretative limitation of our study is related to the heterogeneity of treatment. The cohort included TOF, BAR, UPA, and FIL, with a non‑uniform distribution and different proportions of bDMARD‑naïve patients. This feature is relevant because efficacy, retention and safety may differ across individual molecules and according to line of treatment, whereas our work analyzed all drugs pooled together. Our sample size did not allow any robust analyses for individual JAKis in regard to retention/discontinuation data by drug, and drug‑specific inferences. The number of patients available for analysis progressively decreased during follow-up, reflecting treatment discontinuations, variable follow-up duration, and the real-world nature of data collection. Although repeated-measures analyses allowed the inclusion of all available observations, no formal sensitivity analyses were performed to assess the potential impact of attrition on the study findings. In addition, herpes zoster vaccination status was not systematically available for the entire cohort, as the study period largely preceded the implementation of specific vaccination recommendations for patients receiving JAKis. Consequently, vaccination status was retrospectively assessed only in patients who developed herpes zoster, limiting the ability to evaluate the impact of vaccination on infection risk across the overall study population. Finally, although the definition of cardiometabolic multimorbidity was consistent with previous literature, the exclusion of obesity and previous MACE may have underestimated certain aspects of cardiometabolic burden, and alternative definitions could identify different patient subsets to be fully addressed in future studies.

In conclusion, in our “real-life” multicenter cohort in patients with RA, JAKis demonstrated effectiveness with an acceptable safety profile despite the relatively short follow-up. Although the lack of power to rule out clinically relevant differences for rare events, a low incidence of AEs of special interest was observed, including MACE and malignancies, without venous thromboembolism, and serious infections. The presence of cardiometabolic multimorbidity, T2D, and age ≥ 65 years did not appear to negatively influence treatment response or safety of JAKis. Surely, larger studies with longer follow‑up and dedicated correlation analyses are needed to fully confirm and extend these findings.

## Data Availability

All data relevant to the study are included in the article.
